# Validation of the Argentine version of the epistemic trust, mistrust, and credulity questionnaire

**DOI:** 10.1371/journal.pone.0311352

**Published:** 2024-10-03

**Authors:** Andrea Rodriguez Quiroga, Juan Segundo Peña Loray, Laura Bongiardino, María Eugenia Malleville, Laura Borensztein, Arantxa Y. Arredondo, Antonia Najas-García, Saskia Ivana Aufenacker, Camila Yosa, María Sol Asencio, Milagros Guido, Marianne Cottin, Camila Botero

**Affiliations:** 1 Fundación Turning Point para la Salud y la Sostenibilidad, Barcelona, España; 2 Escuela de Psicología, Universidad Finis Terrae, Santiago de Chile, Chile; Instituto Mexicano del Seguro Social, MEXICO

## Abstract

Epistemic trust refers to the trust in communicated knowledge, specifically an individual’s ability to regard knowledge conveyed by others as meaningful, relevant to oneself, and applicable to other contexts. This area has received considerable attention in recent psychological literature, though predominantly from a theoretical perspective. The main objective of this study was to test the factorial validity of the Epistemic Trust, Mistrust, and Credulity Questionnaire (ETMCQ) on an Argentine setting. Based on two studies (Study 1, *n* = 1018; Study 2, *n* = 559), the factorial structure of the instrument and its internal consistency were examined (S1 Appendix). In the second study, the factorial structure was confirmed, test-retest reliability was analysed, and associations between epistemic stances and sociodemographic variables, hypomentalisation, attachment styles, childhood traumatic experiences, and anxious-depressive symptomatology were explored. A satisfactory three-factor solution with 15 items and residual correlations was found in both studies, with stable scores over time. Significant positive correlations were found with anxious and fearful-avoidant attachment, hypomentalisation, childhood traumatic experiences, and psychopathological symptomatology. Post-hoc analysis revealed that, on the one hand, gender acts as a moderator in the relationship between hypomentalisation and epistemic mistrust. On the other hand, economic level and educational level moderate the relationship between hypomentalisation and epistemic credulity. Measurement invariance across gender was tested and found satisfactory, with significant differences subsequently observed in the epistemic trust factor. In conclusion, the Argentine version of the ETMCQ provides an empirical measure for use in non-clinical samples. Its application could facilitate clinically and theoretically relevant findings.

## Introduction

The concept of Epistemic trust (ET) is complex and has been defined in various ways. It is broadly understood as the trust that individuals have in communicated knowledge [1]. In this research, we understand ET as the particular trust in the value of social knowledge, in other words, a person’s ability to view information shared by others as meaningful, personally relevant, and applicable in different situations [2–4].

While rooted in disciplines such as epistemology and philosophy [5–7], the ET construct has recently been incorporated into the corpus of empirical-theoretical explorations to help understand the processes underlying different psychopathological manifestations [3,8,9]. The latter are supported by research conducted by developmental psychologists, Csibra and Gergely [10], according to which humans are argued to possess a filter mechanism for selecting knowledge and that interpersonal cues are crucial in the effective learning of shared cultural behaviours [11]. Individuals exhibit sensitivity to specific cues that signal the importance and relevance of communications [9]. Developmentally, interpersonal relationships between caregivers and infants are shaped by the infants’ sensitivity to the communicator’s “ostensive cues” (demonstrations by the communicator of the intention to convey new and relevant information, such as different tones of voice or eye contact), which serve as indicators that the information about to be conveyed is reliable and personally relevant to the child [12]. These cues are acknowledged as agentive, and unique to individuals, making the recipient open to transferred knowledge [9] and can be considered as triggers that open communication channels for cultural knowledge, thereby facilitating the effective learning of shared cultural information by encoding it as relevant, generalizable, and important for the recipient [13].

Based on these notions [10] and together with the concept of epistemic vigilance developed by Sperber et al. [1] (in reference to the cognitive mechanism that allows the assessment of the reliability of information from others), Fonagy and Allison [8] proposed a theoretical model of ET in which it is fundamental to explain how human beings transmit and acquire social and relational knowledge, linking this construct to the intergenerational transmission of attachment and mentalisation. In caregiver-child relationships marked by a secure attachment, the interaction and communication between the two allows the latter to simultaneously decrease the level of epistemic vigilance and open the ET channel. In this same context of security in the attachment relationship, the interpersonal exchange marked by ostensive signals facilitates and promotes the development of the mentalisation capacity, as it enables the child to perceive themself as distinct from the caregiver and as an agent in the relationship [14]. This epistemic stance supports the capacity for social learning, allowing the individual to benefit from their social environment and creating a process of salutogenesis [3]. In other words, the epistemic stance characterised by trust in socially transmitted knowledge is interrelated with both the attachment experience and development of mentalising skills.

According to Luyten et al. [15], the epistemic stance characterised by trust may be impaired by experiences of physical/emotional abuse or neglect in the context of the attachment relationship. Due to the particularly harmful nature of such experiences it may generate epistemic petrification and/or loss of adequate vigilance [2]; in other words these adverse experiences may result in the individual adopting another type of epistemic stance, namely Epistemic Mistrust (EM) or Epistemic Credulity (EC). In the first case, the stance towards transmitted information presents aspects of rigidity and hypervigilance, manifesting itself as a tendency to treat any source of information as unreliable or malicious. In the second case, the ability to discriminate between the information received by others is altered, manifesting itself in an excessive trust in others and the increased vulnerability of the individual to be manipulated and/or mistreated by others. These last two dimensions (EM and EC) may hinder the adaptive response capacity to certain difficulties and challenges, which interferes with the effectiveness of psychotherapy and increases the risk of developing psychopathological symptomatology [3,15]. In fact, Campbell et al. [2] supports the fact that EM and EC are variables of interest in relation to the severity of global psychopathology, as both factors partially explain the link between early adversity and mental health symptoms. However, trust did not influence the relationship between childhood traumatic experiences and mental health symptoms [2]. Other research suggests that specifically the adoption of an epistemic stance characterised by ET may function as a protective factor against the development and manifestation of various psychopathological conditions [3,15]. The lack of consensus in the results highlights the need to corroborate the factors of ET in relation to psychological symptomatology, which deserves further investigation.

In the psychotherapeutic setting, Fonagy and Allison [8] have proposed that an acceptable level of ET within the patient-therapist interaction is of crucial importance for the success of psychotherapeutic interventions. In short, the type of epistemic stance adopted towards the transmitted information (be it Trust, Mistrust or Credulity), may play a central role in the development of psychopathological symptomatology and in the outcome of the psychotherapeutic process. Therefore, the restoration of adequate levels of ET can be considered as a central goal of psychotherapy [4]. As already noted, the constructs of ET, EM, and EC play a central role in the psychotherapeutic process and, particularly, in the design and implementation of effective and efficient psychotherapeutic interventions. In this sense, expanding empirical knowledge about ET -a transtheoretical, transdiagnostic, and dynamic construct- would contribute to future research on the effectiveness of different forms of psychotherapy.

Considering the role it has in the process and results of psychotherapy, it is particularly important to have valid and reliable tools for the operationalisation and measurement of the epistemic stance in all its dimensions, as it could favourably contribute to understanding how each individual assimilates and shares information considered relevant to themselves, as well as how cultural and relational knowledge is transmitted. Most of the research on the assessment and measurement of ETMCQ factors comes from WEIRD (Western, Educated, Industrialised, Wealthy and Democratic) countries, characterised by individualism, personal achievement ethic, mistrust of authority and universal justice orientation, amongst others [16]. Therefore, there is a need to investigate the characteristics of the construct in other non-WEIRD countries and to adapt the ETMCQ in light of other cultures [17].

To this end, Campbell et al. [2] have carried out the development and validation of the the Epistemic Trust, Mistrust and Credulity Questionnaire (ETMCQ), which represents the first instrument developed for the measurement of ET and its dimensions. The questionnaire consists of a 15-item self-report measure designed to assess the various forms of epistemic stances (ET, EM, EC). Regarding the factorial validity of the instrument, the authors found a three-factor internal structure through exploratory and confirmatory factor analyses, which is empirically consistent with the hypothesis of the existence of the three epistemic stances (Trust, Mistrust and Credulity), based on Grice’s [18] and relevance theories [19]. Strong indicators of fit and reliability were also found. In relation to convergent and divergent validity, significant positive associations were found between scores on the EM and EC subscales with low mentalisation, adverse childhood experiences, psychopathological symptoms, and insecure and avoidant attachments.

Several studies have analysed the factor structure of the ETMCQ, all of them identifying a three-factor structure, although with differences in the number and distribution of items. For example, in an Italian sample [20] they reported a 14-item solution, eliminating item 11 corresponding to the EC factor and grouping item 15 to the mistrust factor. In the French validation [21], a 12-item solution was reported, eliminating items 1, 3 and 6, but maintaining the original distribution of the factors according to the original author. An Iranian sample [17] also reported a 14-item solution, eliminating item 14, corresponding to the mistrust factor, and retaining the distribution of the items according to the original author. These studies have also demonstrated the criterion validity of the ETMCQ when applied to psychological variables in large representative populations. The authors of the Iran study cited above [17] had clarified that this criterion validity only occurs with EM and EC. These findings provide additional evidence supporting key assumptions about the relationships between dysfunctional epistemic stances and adverse childhood experiences, as well as insecure attachment, poor mentalisation, and psychopathology.

The main objective of the presented research was to validate the ETMCQ version in a non-clinical Argentinean sample. Specifically, it was proposed:

1. To test the factorial structure of the instrument (using Confirmatory Factor Analysis).
2. To evaluate the internal consistency and reliability over time of the ETMCQ, through Cronbach’s Alpha and the Intraclass Correlation Coefficient (ICC) test-retest, respectively.
3. To analyse the relationship between ET, EM and EC and sociodemographic variables.
4. To explore the associations between ET, EM, EC and mentalisation, attachment styles, adverse childhood experiences and the presence of anxious-depressive symptomatology, similar to the one previously proposed by Campbell et al. [2].
5. To test measures of gender invariance and explore differences in ET, EM and EC levels between genders.

The hypotheses for the studies were:

1. The Argentine version of the ETMCQ would have an internal structure of 3 factors (ET, EM and EC) similar to the one previously proposed by Campbell et al. [2].
2. Higher levels of ET would be negatively associated with the presence of anxious-depressive symptomatology, anxious and fearful avoidant attachment styles, and adverse childhood experiences, similar to that previously proposed by Campbell et al. [2].
3. Both EM and EC would be associated with a higher rate of adverse childhood experiences, such as abuse, maltreatment and/or neglect, with higher levels of hypomentalisation, anxiety and depressive symptoms, and anxious and fearful avoidant attachment styles.
4. Significant differences are expected between genders in the scores of the three factors (ET, EM and EC).

## Materials and methods

This research, with a multiple quantitative design and a correlational explanatory non-experimental scope, consisted of two studies. The first aimed to explore the structure of the instrument, and the second to explore the criterion validity and confirm the model.

### Participants

#### Study 1

In the first study, the sample consisted of 1018 participants who completed the ETMCQ and an ad hoc questionnaire (Study 1, *n* = 1018), of whom 582 were women (57.17%), aged between 18 and 89 years (*M* = 40.99, *SD* = 17.02).

#### Study 2

*Test*. The first part of study 2 involved 559 participants, aged between 18 and 80 years (*M* = 42.77, *SD* = 14.04), of whom 271 identified themselves as women (48.48%) (see Table 1). We inquired about the province of residence. Most of the participants resided in the province of Buenos Aires (*n* = 217, 38.82%), followed by the Autonomous City of Buenos Aires (*n* = 125, 22.36%), Córdoba (*n* = 39, 6.98%), Santa Fe (*n* = 39, 6.98%), Mendoza (*n* = 23, 4.11%), Entre Ríos (*n* = 17, 3.04%), Tucumán (*n* = 17, 3.04%), Chaco (*n* = 13, 2.33%), Corrientes (*n* = 12, 2.15%), Santiago del Estero (*n* = 7, 1.26%), Misiones (*n* = 6, 1.07%), Río Negro (*n* = 6, 1.07%), Jujuy, Salta, San Juan and San Luis (*n* = 5, 0.89% each), Chubut (*n* = 4, 0.72), Catamarca, La Pampa, La Rioja, Neuquén (*n* = 3, 0.54% each), Formosa, and Tierra del Fuego, Antarctica and South Atlantic Islands (*n* = 1, 0.18%). Finally, the only province from which we did not receive a response was Santa Cruz.

**Table 1:** Descriptive information of the demographic variables of the Argentine sample (Study 1 & 2).

**Gender**						
		Female	Male	Non-Binary	Other/ Prefer not to say	
Study 1		582 (57.17%)	436 (42.83%)	0	0	
Study 2	Test	271 (48.48%)	287 (51.33%)	1 (0.18%)	0	
	Retest	137 (44.92%)	167 (54.75%)	1 (0.33%)	0	
**Age**						
		18-29	30-39	40-49	50-59	>60
Study 1		340 (33.40%)	171 (16.80%)	174 (17.09%)	156 (15.32%)	177 (17.39%)
Study 2	Test	114 (20.39%)	138 (24.69%)	121 (21.65%)	112 (20.04%)	74 (13.24%)
	Retest	60 (19.67%)	84 (27.54%)	68 (22.30%)	63 (20.66%)	30 (9.84%)
**Marital Status**						
		Single	In a relationship/ civil/ married	Separated/ Divorced	Widowed	Prefer not to say
Study 1		517 (50.79%)	342 (33.60%)	118 (11.59%)	41 (4.03%)	0
Study 2	Test	178 (31.84%)	326 (58.32%)	41 (7.33%)	14 (2.50%)	0
	Retest	94 (30.82%)	176 (57.70%)	27 (8.85%)	8 (2.62%)	0
**Economic level**						
		Low	Lower middle	Middle	Upper middle	High
Study 1		24 (2.36%)	104 (10.22%)	577 (56.68%)	269 (26.42%)	44 (4.32%)
Study 2	Test	28 (5%)	117 (20.93%)	313 (55.99%)	92 (16.46%)	9 (1.61%)
	Retest	12 (3.93%)	65 (21.31%)	174 (57.05%)	48 (15.73%)	6 (1.97%)
**Perceived economic sufficiency**						
		The money is enough for him/her and he/she can save a little.	The money is enough for them, but they cannot save.	The money is not enough for them.	I prefer not to mention.	
Study 1		-	-	-	-	
Study 2	Test	245 (43.83%)	240 (42.93%)	72 (12.88%)	2 (0.36%)	
	Retest	127 (41.64%)	146 (47.87%)	31 (10.16%)	1 (0.33%)	
**Education Level**						
		Primary	Secondary	University degree/ tertiary studies.	Postgraduate	Prefer not to say
Study 1		-	-	-	-	-
Study 2	Test	6 (1.07%)	158 (28.26%)	355 (63.51%)	39 (6.98%)	1 (0.18%)
	Retest	3 (1%)	86 (28.2%)	197 (64.6%)	17 (5.6%)	2 (0.65%)
**Current work status**						
		Full time	Part time	Self employed	Student	Retired
Study 1		-	-	-	-	-
Study 2	Test	309 (55.28%)	78 (13.95%)	83 (14.85%)	28 (5%)	33 (5.90%)
	Retest	181 (59.34%)	43 (14.1%)	40 (13.11%)	10 (3.28%)	15 (4.92%)
		Home keeper/not working or seeking	On furlough	Prefer not to say		
Study 1		-	-	-		
Study 2	Test	16 (2.86%)	4 (0.72%)	8 (1.43%)		
	Retest	12 (3.93%)	4 (1.31%)	0		
**Urban setting**						
		Urban	Rural	Other/ Prefer not to say		
Study 1		-	-	-		
Study 2	Test	531 (94.99%)	21 (3.76%)	7 (1.25%)		
	Retest	291 (95.41%)	11 (3.61%)	3 (0.98%)		
**Ethnicity**						
		White	Asian or Asian British	Black/ African/ Caribbean/ Black British	Mixed/ Multiple ethnic groups	Other/ Prefer not to say
Study 1		-	-	-	-	-
Study 2	Test	448 (80.14%)	2 (0.36%)	8 (1.43%)	84 (15.03%)	17 (3.04%)
	Retest	248 (81.31%)	0	5 (1.64%)	41 (13.44%)	11 (3.61%)

#### Retest

In the second part of study 2, 305 participants participated and responded for the second time to the Argentine version of the ETMCQ scale. The mean age of the participants was 41.45 years (*SD* = 12.95), with a minimum age of 18 years and a maximum age of 72 years (see Table 1). Most participants resided in the province of Buenos Aires (*n* = 123, 40.3%), followed by the Autonomous City of Buenos Aires (*n* = 66, 21.6%). Other places of residence included Córdoba (*n* = 25, 8.2%), Santa Fe (*n* = 19, 6.2%), Chaco (*n* = 10, 3.3%), Mendoza and Tucumán (*n* = 9, 3% each), Corrientes (*n* = 8, 2.6%), Entre Ríos (*n* = 7, 2.3%), Misiones (*n* = 4, 1.3%), Jujuy, Salta, San Juan, San Luis, and Santiago del Estero (*n* = 3, 1% each), Chubut, Neuquén, and Río Negro (*n* = 2, 0.7% each), Catamarca, Formosa, La Pampa and La Rioja (*n* = 1, 0.3% each). No responses were obtained from Santa Cruz and Tierra del Fuego, Antarctica and South Atlantic Islands.

### Instruments

**The Epistemic Trust, Mistrust and Credulity Questionnaire (ETMCQ) [2]**, is a self-report instrument composed of 15 items that evaluates through three factors the epistemic stances: 5 items of Trust; 5 items of Mistrust, and 5 items of Credulity, towards the information received from others. Some of its items are: *“1*. *I usually ask for advice from people when I have a personal problem”* [*“1*. *Usualmente pido consejo a la gente cuando tengo un problema personal”*] (ET), *“3*. *I prefer to find out things on my own on the internet rather than having to ask other people for information”* [*“3*. *Prefiero averiguar por mi cuenta las cosas en internet a tener que pedirle información a otras personas”*] (EM), *“5*. *I am often considered naïve because I believe almost everything I am told”* [*“5*. *Con frecuencia me consideran ingenuo/a porque creo casi todo lo que me dicen”*] (EC). These items are evaluated on a 7-point Likert scale (1 = “Strongly disagree” to 7 = “Strongly agree”). Higher scores indicate higher levels of trust, mistrust or credulity respectively. The reliability reported by the original author [2] for the total scale was α = 0.78, for ET α = 0.76, for EM α = 0.72 and for EC α = 0.81. The reliability reported for study 1 for the total scale was α = 0.71 and ω = 0.73, for ET α = 0.73 and ω = 0.77, for EM α = 0.63 and ω = 0.66, and for EC α = 0.72 and ω = 0.79. For study 2, the total scale value was α = 0.81 and ω = 0.85 for ET α = 0.77 and ω = 0.81, for EM α = 0.65 and ω = 0.68, and for EC α = 0.78 and ω = 0.84. The Chilean version [22] of the instrument was used as a basis, making a linguistic and cultural adaptation for the validation of the Argentine version. The Argentine version of the ETMCQ is available in the supplementary information section of this study.

**The Reflective Functioning Questionnaire (RFQ-8) [23]** is a self-report instrument developed to assess difficulties in mentalisation. The Argentine version of the RFQ validated by Rodriguez Quiroga et al. [24] was used, which presents a unidimensional structure that captures hypomentalisation. Higher scores indicate greater difficulties in mentalising, while lower scores indicate better mentalising abilities. It consists of 8 items, such as: “2. I don’t always know why I do what I do”. These items are evaluated on a 7-point Likert scale (1 = “Strongly disagree”, 7 = “Strongly agree”). The reliability reported by the original author [23] for RFQ_U and RFQ_C were α = 0.77 and α = 0.65 in the clinical sample, and α = 0.63 and α = 0.67 in the non-clinical sample, and the reliability reported by the author of the adapted scale [24] was α = 0.7. In the present investigation we obtained a value of α = 0.81 and ω = 0.86.

**The Argentine attachment styles scale [25]** is a self-report psychometric instrument that assesses attachment in two different contexts: romantic and non-romantic dyads, using a 4-point Likert scale (1 = “Almost never”, 4 = “Almost always”). The non-romantic scale was chosen, which is composed of 11 items, initially assessed in four dimensions: secure attachment, anxious attachment, avoidant attachment and fearful attachment. The original author merged the last two, leaving a total of three dimensions. In this study only anxious attachment (3 items) and fearful-avoidant attachment (6 items) were administered, similarly to Campbell et al. [2]. This corresponds to a total of 9 items, for example: “8. Engaging in affective relationships scares me”. Higher scores indicate greater anxious attachment or fearful-avoidant attachment. The reliability reported by the original author [25] was α = 0.42 for anxious attachment and 0.60 for fearful-avoidant attachment. In the present investigation we obtained a value of α = 0.65 and ω = .66 for anxious attachment dimension and α = 0.79 and ω = .82 for fearful-avoidant attachment dimension.

**The Hopkins Symptom Checklist (HSCL-11)** [26] is an 11-item instrument that assesses the type of symptomatology (mainly anxiety and depression symptoms) experienced in the last 7 days, such as “6. Lack of hope for the future”, using a 4-option Likert scale (1 = “Not at all”, 4 = “Very much”). The Argentine version of the HSCL-11, validated by Gómez-Penedo et al. [27], was used for the present study. The higher the score, the greater the symptomatology experienced in the last 7 days. The reliability reported by the original author [26] was α = 0.85, and the reliability reported by the author of the adapted scale [27] was α = 0.81. In the present investigation we obtained a value of α = 0.89 and ω = 0.91.

**The brief version of the Child Trauma Questionnaire (CTQ-SF)** [28] is a retrospective self-report instrument that assesses various types of child abuse and maltreatment. The Chilean version of the CTQ-SF [29] was used due to the lack of validated scales in Argentina to measure the construct, and because of the cultural and linguistic proximity between the Chilean and Argentine populations. It consists of 23 items, such as “5. Someone in my family made me feel important or special”, in the following 5 factors: 5 emotional abuse (EA) items, 5 physical abuse (PA) items, 5 sexual abuse (SA) items, 5 emotional neglect (NE) items, and 3 physical neglect (PN) items, assessed with 5-point Likert-type responses (1 = “Never”, 5 = “Almost always”). High scores account for possible greater child trauma, while low scores denote less child trauma. The reliability reported by the original author [28] was α = 0.87 for emotional abuse, α = 0.84 for physical abuse, α = 0.94 for sexual abuse, α = 0.88 for emotional neglect, α = 0.69 for physical neglect. The author’s reported reliability of the adapted scale [29] was α = 0.85 for emotional abuse, α = 0.87 for physical abuse, α = 0.93 for sexual abuse, α = 0.79 for emotional neglect, and α = 0.79 for physical neglect. In the present investigation we obtained a value of α = 0.89 and ω = 0.90 for emotional abuse, α = 0.90 and ω = 0.93 for physical abuse, α = 0.92 and ω = 0.95 for sexual abuse, α = 0.86 and ω = 0.90 for emotional neglect and α = 0.61 and ω = 0.63 for physical neglect.

### Procedures

To validate the original version of the ETMCQ in Argentina, the existence of previous versions adapted to the Spanish language was investigated, leading to the finding of the translation made by Becerra et al. [22], validated for Chile. This Chilean version began with a back-translation into English by four independent translators. To verify the accuracy of the initial translation, both versions were compared. Subsequently, these translations were subjected to the exhaustive review of five experts, in order to ensure that the items remained faithful to the original.

For the validation of the Argentine version corresponding to this study, the work of Becerra et al. [22] was revisited, and the ETMCQ items were evaluated by 9 Argentine experts to identify changes that merit the scale, with items 7 and 9 being modified (the suffix "/a" was added to the words "myself" [“mismo”] and "hurt" [“herido”] respectively), and item 14 was also syntactically modified in a single word according to the usage of the language in Argentina ("sean" replaced by "son").

Study data were collected through an online survey using the SurveyMonkey platform.

#### Study 1

In the first study, participants gave their consent and completed a questionnaire that included the Argentine version of the ETMCQ and an ad hoc questionnaire for the collection of sociodemographic data: age, gender, marital status and economic level. Participants were recruited through snowball sampling, via mailing lists and social media channels (e.g., by spreading the survey through WhatsApp, Facebook and Instagram profiles). The survey was conducted online from September 9 to October 24, 2022.

A total of 1303 questionnaire responses were collected. When applying the inclusion criteria, which required participants to be 18 years of age or older, 3 questionnaires from minors and 78 from non-Argentine participants were eliminated. In addition, 204 questionnaires with incomplete responses on at least one item were discarded, leaving a total of 1018 questionnaires for analysis. Participants were not rewarded.

#### Study 2

The second study was divided into two parts, the first aimed at remeasuring the ETMCQ scale along with additional self-report measures, intended to investigate the relationship between ETMCQ scores and other psychological dimensions, such as reflective functioning, adverse childhood experiences, attachment, and symptoms of psychopathology. Data collection for this second study (test and retest) was carried out through a company specialised in digital surveys. In addition to providing informed consent, participants in both instances also completed an ad hoc questionnaire designed to collect sociodemographic data: age, gender, educational level. In addition, we included data on marital status, economic level, current employment status, province of residence in Argentina, living environment (rural, urban or other), ethnic group and perceived economic sufficiency (a person’s subjective perception of whether their income and financial resources are sufficient to cover their needs).

*Inclusion criteria*. Participants were required to be 18 years of age or older and residents of Argentina.

*Test*. A total of 622 questionnaires were collected. Two questionnaires corresponding to minors and 12 questionnaires with incomplete responses in at least one item of any of the scales evaluated were eliminated. In order to comply with the established age group quotas and as a quality measure, 5 questionnaires were eliminated from those participants in which the numerical age response did not coincide (*How old are you*?) [*“¿Qué edad tenés*?*”*] with the response about the age category (*“In addition*, *please select your age group”*) [*“Además*, *te pedimos que selecciones a qué grupo de edad perteneces”*]. In addition, participation was restricted to only one questionnaire per device, and it was verified that there were no duplicate responses from the same IP address, resulting in the elimination of 44 questionnaires. Consequently, the final test sample consisted of 559 participants.

*Retest*. A total of 310 questionnaires were collected. We eliminated 1 questionnaire that belonged to a person who had not participated in the first part (test), and 4 questionnaires that had incomplete answers in at least one item. We verified that all the sociodemographic data provided coincided exactly with those of the first part: age, age group, gender, educational level, marital status, economic level, current employment status, province of residence in Argentina, living environment (rural, urban or other), perceived economic sufficiency and ethnic group. We also checked for duplicate IP addresses. With no inconsistencies found, the final retest sample consisted of 305 participants.

Unlike Study 1, participants in Study 2 received financial compensation for their participation. This compensation was facilitated by a third-party company specialized in data collection through large, diverse respondent panels. The company’s role was strictly limited to recruiting participants and administering the surveys, with no involvement in the study’s design, analysis, or interpretation. This second study was conducted from July 1 to July 17, 2024.

In both studies, all respondents were guaranteed confidentiality. Participants could decide to discontinue the survey at any time and resume it later, or revoke their participation altogether. All procedures performed in this study complied with the standards of the Declaration of Helsinki and its subsequent amendments. The ethics committee of the University Institute of Mental Health (IUSAM) of the APdeBA (Buenos Aires Psychoanalytic Association) considered exempting this study from evaluation, which means that it would not have been subject to review by an ethics committee before its implementation.

### Data analysis

All analyses were carried out using the open source software R version 4.4.1 (2024-06-14) [30], specifically using the packages dyplr version 1.1.4 [31], lavaan version 0.6.18 [32], tidyverse version 2.0.0 [33], haven version 2.5.4 [34], psych version 2.4.6.26 [35], corrplot version 0.92 [36], FactoMineR version 2.11 [37], and semPlot version 1.1.6 [38].

#### Study 1

To evaluate the internal structure of the instrument, a Confirmatory Factor Analysis (CFA) was performed using the Robust Maximum Likelihood estimator (MLR). Three models were considered: 1) a 15-item unidimensional model; 2) a 15-item trifactorial model proposed by Campbell et al. [2]; and 3) the 15-item trifactorial model proposed by Campbell et al. [2] but correlating residuals. The evaluation of the models was based on the Root Mean Square Error of Approximation (RMSEA) and Standardised Root Mean Square Residual, with acceptable values starting below <0.08 [39,40], as well as on the comparative fit index (CFI) and Tucker-Lewis Index (TLI), and values equal to or greater than 0.90 were considered acceptable [41]. In addition, the chi-square value was evaluated to determine the model fit. The best-fit CFA model was also constructed using the test sample from Study 2.

To evaluate the internal consistency of the total scale and the factors in both studies, Cronbach’s alpha coefficient was used, considering adequate values to be above 0.70 [42]. Lower values represent questionable internal consistency [43].

Finally, a gender invariance analysis of the instrument was carried out and the differences in the ETMCQ factor scores according to the gender variable were explored using Student’s t-test.

#### Study 2

In order to confirm the satisfactory model of Study 1 in this second sample, a confirmatory factor analysis (CFA) was performed. Correlational analyses of ETMCQ with demographic characteristics (e.g., age group, economic level, educational level, and perceived economic sufficiency) were performed using Spearman’s Rho statistic. In the analysis of the criterion validity, Pearson correlation coefficients were calculated between the factors of ET, EM and EC, and variables such as age, reflective function (RFQ-8), attachment styles (Attachment Scale), anxious-depressive symptomatology (HSCL-11) and adverse childhood experiences (CTQ-SF). The results of these correlation analyses were corrected for multiple comparisons using a false discovery rate (FDR) of 5%, based on the sequential FDR correction algorithm [44]. Correlation coefficients were evaluated as proposed by de-Winter et al. [45] in psychological assessment: Pearson correlation coefficients will be considered, being 0.25 moderate, 0.5 strong and 0.8 very strong.

The test-retest Intraclass Correlation Coefficient (ICC) was used to assess reliability over time. Values from 0.50 to 0.75 indicate moderate reliability, 0.75 to 0.90 indicate good reliability, and values above 0.90 indicate optimal reliability.

Finally, post-hoc regression analyses were conducted to explore how sociodemographic variables moderate the relationship between hypomentalisation and the three factors of the scale.

## Results

### Study 1

**Fig 1:**
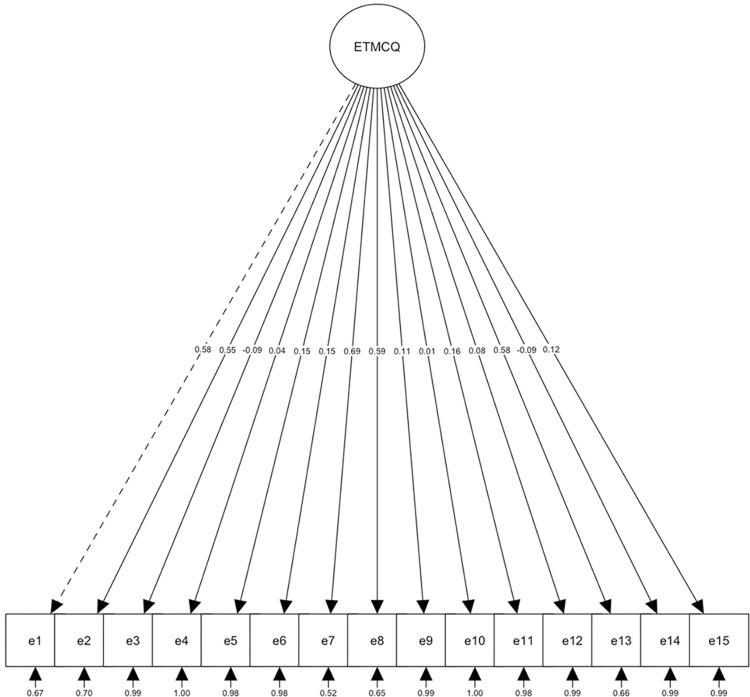
One dimensional model structure.

**Fig 2:**
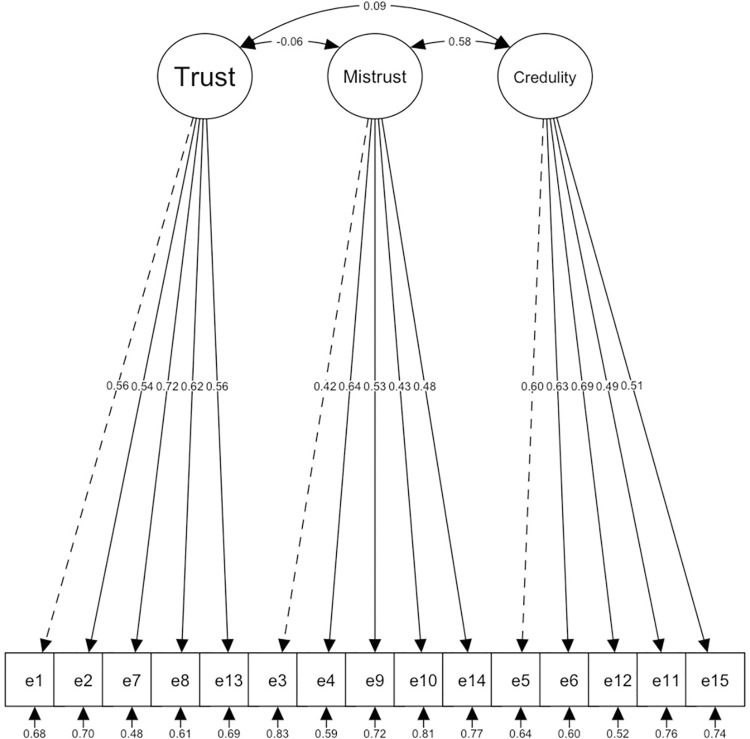
Original 3–factor model structure proposed by Campbell et al. [2].

With the total sample of Study 1, we first performed a Confirmatory Factor Analysis (CFA) in which both a one-factor model (Fig 1 and Table 2) and the original 3-factor, 15-item model proposed by Campbell et al. [2] were analysed without correlating residuals (Fig 2 and Table 2). Both models indicated insufficient fit.

**Table 2:** Goodness–of–fit indexes for the four models.

Model	*χ* ^2^	*df*	*p*	CFI	TLI	RMSEA (90% CI)	SRMR
Unidimensional	2112.121	90	< .01	0.33	0.22	0.15 (0.14–0.15)	0.16
3 factors—15 items Campbell et al. [2]	635.396	87	< .01	0.82	0.78	0.08 (0.07–0.08)	0.07
3 factors—15 items Campbell et al. [2], CRs	292.979	82	< .01	0.93	0.91	0.05 (0.04–0.06)	0.05

*Note*. *χ*^*2*^, Chi squared; *df*, degrees of freedom; *p*, p–value; CFI, Comparative Fit Index; RMSEA, Root Mean Square Error of Approximation; CI, Confidence Interval; SRMR, Standardised Root Mean Square Residual; CRs, Correlated residuals.

**Fig 3:**
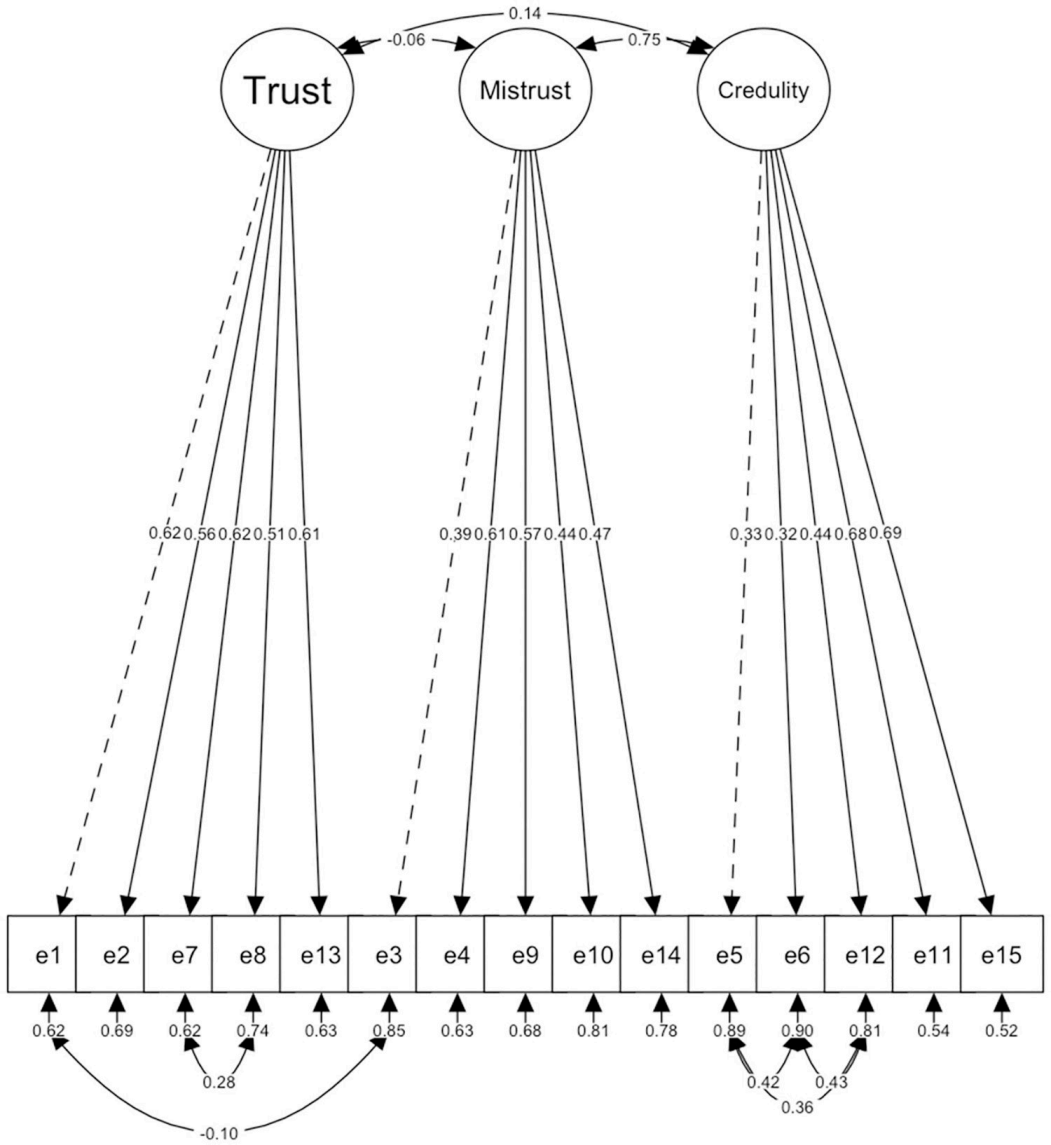
Original 3–factor model structure proposed by Campbell et al. [2], CRs.

Therefore, a third factorial solution of the original model was tested, but correlating residuals among several items with similar content and/or wording proposed by the original author (7 and 8; 3 and 1; 5 and 6; 6 and 12; 5 and 12). All items had loadings greater than 0.30 for each factor (Fig 3 and Table 2), demonstrating a satisfactory fit with all items of the scale. This model fitted correctly. ET and EC factors had a significant but weak positive correlation (*r* = 0.09; *p* = 0.003), and EC factors had a significant and moderate positive correlation (*r* = 0.28; *p* < 0.001). However, the correlation between ET and EM did not reach statistical significance (*r* = -0.04; *p* = 0.24).

#### Study 2

A CFA was performed with the confirmed model from Study 1 using the 15 items and correlating the residuals between the same pairs of items (Model 3) in the Study 2 sample (*n* = 559). The results indicated a satisfactory model fit: χ2 (82) = 261.931; *p* < 00.01, CFI = 0.92, TIL = 0.90, SRMR = 0.05 y RMSEA = 0.06 (IC = 0.05–0.07).

#### Internal consistency (Study 1 & 2)

For the Study 1 sample, the Cronbach’s alpha value indicated acceptable reliability for the full scale (α = 0.71), as well as for the ET (α = 0.73) and EC (α = 0.72) subscales, however, the EM factor obtained a questionable value (α = 0.63). For the Study 2 sample, similar scores were found, with adequate internal consistency for the full scale (α = 0.81), as well as for the ET (α = 0.77) and EC (α = 0.78) factors and again questionable for the EM factor (α = 0.65) (see Table 3).

**Table 3:** Psychometric properties of the items and internal consistency of the epistemic stances (Study 1 & Study 2).

Study 1									Study 2				
Factors	Item	*M* (*SD*)	Citc	Skewness	Kurtosis	α – item	Total α (95% CI)	Total *M* (*SD*)	*M* (*SD*)	Citc	α – item	Total α (95% CI)	Total *M* (*SD*)
ET	item 1	4.5 (1.8)	0.71	-0.43	-0.91	0.7	0.73	5.1 (1.1)	4.2 (1.7)	0.73	0.74	0.77	5 (1.1)
	item 2	5.4 (1.5)	0.67	-1.11	0.60	0.7			5.4 (1.4)	0.73	0.71		
	item 7	5.1 (1.4)	0.73	-0.79	0.27	0.66			4.9 (1.4)	0.74	0.71		
	item 8	5.5 (1.3)	0.67	-1.09	1.03	0.69			5.5 (1.2)	0.69	0.73		
	item 13	5.1 (1.6)	0.70	-0.80	-0.18	0.69			4.8 (1.6)	0.72	0.73		
EM	item 3	3.9 (1.7)	0.62	0.07	-0.86	0.58	0.63	3.8 (1)	5.1 (1.5)	0.59	0.62	0.65	4.4 (1)
	item 4	4.0 (1.7)	0.68	-0.03	-0.88	0.54			4.4 (1.6)	0.7	0.56		
	item 9	4.3 (1.6)	0.64	-0.19	-0.69	0.56			4.6 (1.5)	0.65	0.59		
	item 10	3.4 (1.7)	0.6	0.29	-0.86	0.6			4.0 (1.6)	0.66	0.59		
	item 14	3.5 (1.6)	0.62	0.29	-0.80	0.58			4.0 (1.5)	0.61	0.61		
EC	item 5	3.0 (1.7)	0.69	0.58	-0.75	0.67	0.72	3.2 (1.2)	3.0 (1.7)	0.74	0.74	0.78	3.3 (1.2)
	item 6	2.9 (1.6)	0.69	0.67	-0.47	0.66			2.8 (1.5)	0.72	0.74		
	item 11	3.7 (1.7)	0.63	0.09	-1.00	0.69			4.0 (1.7)	0.72	0.75		
	item 12	2.7 (1.7)	0.75	0.82	-0.45	0.63			2.6 (1.6)	0.78	0.71		
	item 15	3.8 (1.8)	0.67	-0.007	-1.15	0.69			4.0 (1.8)	0.69	0.77		

Note. ET, factor Epistemic Trust; EM, factor Epistemic Mistrust; EC, factor Epistemic Credulity; *M*, median; (*SD*), standard deviation; Citc, corrected total item correlation; α – item, Cronbach’s alpha if the item is dropped; CI, Confidence Interval; α, Cronbach’s alpha.

### Study 2

#### Test-retest reliability

A total of 305 participants (54.56% of the sample from the first taking of the instrument *n* = 559) participated in the retest of study 2 (see Table 1). Intraclass correlation coefficients were calculated (ICC) and their 95% confidence intervals, based on a mixed effects model of absolute agreement and a single rating. The results showed that the consistency between the two intakes for ET was high, with an ICC of 0.67 (95% CI: 0.604–0.728), indicating good reliability of measurements over time, EM showed moderate consistency, with an ICC of 0.53 (95% CI: 0.446–0.608), suggesting acceptable reliability but with increased variability. Finally, the ICC for EC was 0.66 (95% CI: 0.598–0.724), similar to ET, indicating a high consistency in the measurements between the two intakes. All ICCs were significantly different from zero, with *p* values < 0.001 for all three factors (ET, EM and EC).

When examining Spearman correlation coefficients between ETMCQ factors and age in Study 2 (first part *n* = 559), small but significant negative correlations were found between age and ET (Rho = -0.14, *p* < 0.001), between age and EM (Rho = -0.13, *p* < 0.001), and age and EC (Rho = -0.19, *p* < 0.001). In addition, a significant negative correlation was observed between educational level and EM (Rho = -0.11, *p* = 0.04). An inverse correlation was found between ET and perceived economic sufficiency (Rho = -0.09; *p* = 0.05) and positive correlation with EC (Rho = 0.1; *p* = 0.05). Economic level did not show significant correlations with any factor.

The criterion validity analysis was performed by calculating Pearson’s correlation coefficient between the factors of ET, EM and EC, and reflective function (RFQ-8), attachment styles (Attachment Scale), anxious-depressive symptomatology (HSCL-11) and adverse childhood experiences (CTQ-SF). Significant positive correlations were found between ET, EM and EC with hypomentalisation. Emotional, physical, and sexual abuse correlated positively with EM and EC. Emotional and physical neglect were inversely correlated with ET and positively correlated with EM and EC. Anxious-depressive symptomatology was directly correlated with EM and EC (see Table 4).

**Table 4:** Correlations of epistemic stances, hypomentalisation, attachment, childhood trauma, and mental health symptoms (Study 2).

Study 2			
	ET	EM	EC
Reflective Functioning (Hypomentalisation)	0.28***	0.44***	0.59***
Attachment			
Anxious	0.17***	0.40***	0.51***
Avoidant-Insecure	0.05	0.55***	0.37***
Traumatic Experiences			
Emotional Abuse	-0.05	0.20***	0.3***
Physical Abuse	-0.06	0.16***	0.25***
Sexual Abuse	-0.06	0.09*	0.25***
Emotional Negligence	-0.19***	0.10*	0.18***
Physical Negligence	-0.13**	0.14**	0.22***
HSCL-11	0.04	0.28***	0.36***

Note. The scales have been analysed with Pearson’s r.; FDR (False Discovery Rate) has been applied. *p < 0.05

**p < 0.01

***p < 0.001.

### Analysis of sociodemographic variables as moderators of the relationship between hypomentalisation and ET, EM, and EC

In order to further explore the relationship between mentalisation and epistemic stance, a post hoc moderation analysis was conducted to investigate whether the sociodemographic characteristics of the sample moderated the relationship between hypomentalisation and epistemic stance factors. Results showed a significant value for gender as a moderating variable between RFQ and EM (Adjusted *R^2^* = 19,5%, *F*(3, 555) = 46,13, *p < 0*,*001*). The relationship between RFQ and EC was moderated by economic (Adjusted *R^2^* = 34,7%, *F*(3, 555) = 99,83, *p* < 0,001) and academic level (Adjusted *R^2^* = 35,0%, *F*(3, 555) = 101,2, *p* < 0,001) (see Table 5).

**Table 5:** Analysis of sociodemographic variables as moderators of the relationship between hypomentalisation and epistemic stances.

Dependent variable		*β*	*t*
ET	Gender * RFQ	-0.249	*t* = -1.478; *p* = 0.14
	Age * RFQ	0.07	*t* = 0.481; *p* = 0.631
	Socioeconomic level * RFQ	-0.04	*t* = -0.218; *p* = 0.829
	Perceived economic sufficiency * RFQ	0.24	*t* = 1.459; *p* = 0.145
	Education level * RFQ	0.01	*t* = 0.05; *p* = 0.961
EM	Gender * RFQ	-0.312	*t* = –1.986; *p* = 0.05
	Age * RFQ	-0.08	*t* = -0.548; *p* = 0.584
	Socioeconomic level * RFQ	0.11	*t* = 0.650; *p* = 0.516
	Perceived economic sufficiency * RFQ	-0.147	*t = -0*.*942; p = 0*.*347*
	Education level * RFQ	0.307	*t* = 1.548; *p* = 0.122
EC	Gender * RFQ	-0.10	*t* = -0.73; *p* = 0.47
	Age * RFQ	0.075	*t* = 0.584; *p* = 0.56
	Socioeconomic level * RFQ	0.39	*t* = 2.512; *p* = 0.01
	Perceived economic sufficiency * RFQ	-0.063	*t = -0*.*450; p = 0*.*653*
	Education level * RFQ	0.504	*t* = 2.831; *p* = 0.005

*Note*. ET, factor Epistemic Trust; EM, factor Epistemic Mistrust; EC, factor Epistemic Credulity; *β*, standardised regression coefficients*; t*, test.

Table 6 shows the results obtained for measurement invariance across gender. The configural (M1) and metric (M2) invariance showed a good fit, with minimal changes between M1 and M2, indicating metric invariance across gender. The indices for the scalar invariance model (M3) showed good fit, and the comparison between M2 and M3 showed minimal differences, supporting scalar invariance. The results for the strict invariance model (M4) also showed a good fit, with minimal differences between M3 and M4. These results suggest invariance of the measure across gender.

**Table 6:** Gender invariance analysis: Goodness–of–fit indexes and model comparison.

Model	χ2	df	*p*	CFI	TLI	RMSEA (90% CI)	SRMR	|Δ RMSEA|	|Δ SRMR|	|Δ CFI|
M1. Configural	397.464	164	< 0.001	0.92	0.90	0.05	0.05	-	-	-
M2. Metric	409.795	176	< 0.001	0.92	0.906	0.05	0.05	0.001	0.001	< 0.001
M3. Scalar	443.666	188	< 0.001	0.91	0.904	0.05	0.05	< 0.001	0.001	0.007
M4. Strict	461.676	203	< 0.001	0.91	0.910	0.05	0.06	0.001	0.002	0.001

Note. χ2, chi–squared test; df, degrees of freedom; p, p value; CFI, comparative fit index; TLI, Tucker–Lewis index; RMSEA, root mean square error of approximation; CI, confidence interval; SRMR, standardised root mean square residual. Change values compare the model to the previous model and comparison criteria are: ΔCFI<0.010, ΔRMSEA<0.015, ΔSRMR<0.030.

The only significant differences in the ETMCQ factors were found in the ET factor (*t*(906.37) = -6.3641; *p <* .*01*; CI 95% [-0.5607330–0.2964062]; Cohen’s *d* = -0.407), with a higher score for women (*M* = 5.29; *SD* = 1.02) compared to men (*M* = 4.86; *SD* = 1.09). No differences were found in EM (*t*(951.74) = 1.4541; *p = 0*.*146*; CI 95% [-0.03359183 0.22574795]) or EC factors (*t*(985.29) = -1.3491; *p = 0*.*178*; CI 95% [-0.24075671 0.04459038]).

## Discussion and conclusions

The general objective of the present study was to validate the ETMCQ version in a non-clinical Argentine sample. The first specific objective was to determine the factorial structure of the questionnaire. For this purpose, three models were evaluated: unidimensional, three factors with 15 items, and three factors with 15 items and correlating residuals by means of confirmatory factor analysis. The latter model tested confirmed the three-factor structure to be consistent with earlier proposals such as the original scale [2] and in non-clinical samples [17,20,21]. The factor structure was maintained after correlating some residuals, suggesting that although the general model is appropriate, there could be an unexplained variability affecting the representation of the factors. This goes in line with the strategy used by Campbell et al. [2], and Greiner et al. [21]. This confirmatory model was also tested in the study sample 2 with satisfactory results, thereby confirming our first hypothesis. Additionally, positive correlations were found between the dimensions of EM and EC but also between ET and EC, similar to those found in another non-clinical population study [21] and slightly similar to Campbell et al. [2].

The second specific objective was to evaluate the internal consistency and reliability of the questionnaire over time. Regarding the reliability of the scale, the results show that the ET and EC factors present good internal consistency and high test-retest reliability, indicating that these measures are stable and reliable over time. However, the EM factor did not reach acceptable levels of internal consistency and its test-retest reliability, although acceptable, was lower than that of ET and EC. These results are consistent with those reported in a non-WEIRD sample from Iran [17,46].

The low internal consistency of the EM factor suggests that the items may not be uniformly measuring the mistrust construct. In addition, the test-retest reliability score for EM indicates that although this measure scored lower compared to the other two factors, it can be considered stable over time. The fact that the EM results matched those with other studies [17] suggests that there could be contextual or cultural factors influencing the measure. It is important to explore these factors and consider their impact on the validity of the EM factor, such as the cited significant negative correlation between educational level and EM.

The third specific objective was to analyse the relationship between epistemic postures and sociodemographic variables. The results obtained suggest that EM is associated with a lower educational level. Therefore, it is possible to think that this relationship may be complex and not always linear. For example, in Kivimäki’s [47] study, which includes investigation on beliefs about knowledge and its acquisition, epistemic cognition was closely linked to educational attainment, suggesting that more sophisticated epistemic beliefs are generally associated with better academic performance.

Although economic status showed no relationship with any epistemic stance, the perception of the participants’ purchasing power (perceived economic sufficiency) did show a direct relationship with EC and an inverse relationship with trust. The data suggests that more credulous persons might tend to more easily trust the information they receive and to overestimate their economic conditions. However, persons with higher ET may tend to underestimate them. Trust with respect to economic perception has been addressed from different perspectives in the academic literature. For example, a recent study on the perception of perceived economic inequality [48] claims that a negative perception of the economic situation with respect to others is associated with lower levels of social trust.

Regarding the fourth specific objective, positive correlations were found between hypomentalisation and the three factors of the questionnaire. The results showed correlations in EM and EC of moderate to strong magnitude effects. As for ET, the correlation indicated a moderate strength. This finding is similar to those of Campbell et al. [2] who also showed that both EM and EC were associated with difficulties in understanding mental states. However, they did not find a significant relationship with ET. Regarding the positive relationship found between hypomentalisation with ET, persons who understand well the intentions and thoughts of others (low hypomentalisation) might tend to be less naive and gullible in interpersonal relationships. This is because they have a greater ability to assess the trustworthiness of others and to adopt a cautious attitude [15,49]. Trust is based on the ability to mentalise, i.e., to understand the intentions and mental states of others, and persons are more likely to adopt a stance of ET towards someone if they appear to be able to mentalise us [50]. However, it is important to be selective in opening oneself to trust, as indiscriminate openness would not be positive from an evolutionary perspective. Therefore, it is necessary to exercise a form of epistemic vigilance in assessing the trustworthiness of others [50].

Taking into account the direct correlations obtained in both factors (ET and EM), it is important to remember, as Kramer [51] explains, that mistrust is not simply the absence of trust. According to Wicks et al. [52], positive and negative expectations coexist in persons with respect to the behaviours of others. Since trust and mistrust are independent concepts, there may be differentiated elements that increase and decrease trust and mistrust.

This is why trust is a dynamic variable; although there are temporary states of balance in relationships, the most frequent would be the existence of certain tensions inherent to social relationships, where attitudes of trust and mistrust coexist [52,53].

Post hoc analyses performed with the aim of examining whether sociodemographic variables moderated the relationship between hypomentalisation and epistemic stances, yielded results that indicate that only gender acts as a moderator in the relationship between hypomentalisation and EM. This result aligns with previous findings showing differences between men and women in how hypomentalisation relates to epistemic stances. For example, Locati et al. [54] studied a sample of adolescents and found that in females ET mediates the association between RF and psychopathology, whereas in males the associations are independent of psychopathology. In the same line, Campbell et al. [2] found a significant gender difference: women scored significantly higher than men on ET and EC and men scored higher on mistrust. More studies are needed in this regard to reach a deeper understanding about this differential relationship between genders with respect to mentalisation and mistrust.

Similarly to past research, socioeconomic and educational levels were found to moderate the relationship between hypomentalisation and EC. For example Liotti et al. [20] found that ET is influenced by several sociodemographic factors, including educational attainment as both mistrust and EC are associated with traumatic childhood experiences, lower levels of mentalisation and emotional regulation, and this may ultimately indirectly influence the educational context [20]. As mentioned before, socioeconomic status was not significantly related to any epistemic stance, however, it appears that economic level acts as a moderator, suggesting that persons with lower mentalisation capacity may adopt a particular epistemic stance as a function of socioeconomic level. These results, together with the mediating influence shown by educational level, may indicate that participants with greater resources have access to more sophisticated education, which would facilitate higher levels of mentalisation and a less mistrustful epistemic stance. There were no sociodemographic variables that moderated the relationship between ET and hypomentalisation.

Regarding the associations between attachment and epistemic stances, this study found a significant positive relationship between anxious attachment and the factors of trust, EM and EC. These findings contrast with the results of previous studies conducted in different cultural contexts. Campbell et al. [2] identified a significant positive relationship between anxious attachment and the factors of EM and EC, without finding a relationship with trust. Similarly, Liotti et al. [20] observed a positive relationship only with mistrust. Greiner et al. study [21] carried out in France, showed positive relationships between anxious attachment and EM and EC, but a negative relationship with trust. Lastly, the aforementioned research conducted in Iran [17] also found positive relationships with EM and EC. These discrepancies may be due to several reasons. Cultural and contextual differences between the samples of the different studies may significantly influence how the attachment dimensions and the ET, EM and EC factors are manifested and perceived.

The direct association between anxious attachment and ET suggests that, although insecurity in adult attachment is associated with greater cognitive closure [55], persons with anxious attachment may be more flexible in their thinking in contexts where they feel emotionally secure. For example, in relationships where their needs are met, they may be more willing to be open to new ideas and trust the information they receive as a mechanism to regulate their anxiety and maintain closeness. Epistemic petrification [3], or epistemic freezing [56–58] which is present in disorganised attachment [2], is characterised by a tendency to defend existing knowledge structures despite them being incorrect or misdirected, due to a need for validation and emotional support. This may lead those with anxious attachment to rely on specific attachment figures who provide them with information that reinforces their beliefs, resulting in selective and context-dependent ET. However, in situations where attachment figures are responsive and supportive, anxiously attached individuals may feel less threatened and thus more open to new information. This may facilitate greater epistemic confidence, as their sense of emotional safety allows them to explore and accept information that they might otherwise reject.

In addition, evidence found by Campbell et al. [2] exploring the in-depth relationships between epistemic stances and attachment styles found that individuals who are preoccupied (high anxious, low avoidant) have little or no difficulty with trust, indeed, they score similarly to participants who belong to the group contrasted as secure (low anxious and low avoidant). However, they tend to score high on EC, suggesting an overvaluation of communication from others at the expense of epistemic self-efficacy.

Campbell et al. [2] research highlights the connection between EM and EC with anxious and fearful-avoidant attachment styles, as we obtained in our findings. This underscores the relationship between how we approach our beliefs and our ability to form secure attachments. EM is associated with more pronounced avoidance, possibly due to a negative perception of dependence on others. Individuals with a fearful attachment style face a significant epistemic challenge, as they tend to exhibit high EM and EC, making them more likely to accept misinformation.

Positive correlations were found between the various types of childhood trauma and the factors of EM and EC, although with a low effect size, especially in the case of the EM factor. On the other hand, correlations with the EC factor mostly reach a moderate effect. Past research [59,60] have found a close relationship between EM and EC with neglect and abuse. Maltreatment not only disorganises the attachment system [61], but also appears to disrupt the development of mentalisation [50]. According to Fonagy et al. [3], attachment trauma leads to a posture of resistance to receiving crucial information about the social environment and how to manage in it, which erodes ET. When a trauma that disempowers subjectivity and affects the ability to mentalise is experienced, EM crystallises. This prevents the persons from learning from the social context and perpetuating maladaptive relationship patterns that can trigger psychopathological manifestations.

With respect to childhood adversity, the fact that ET showed negative (although weak) correlations with neglect (emotional and physical) but not with different types of abuse, may confirm that neglect and abuse have different developmental trajectories [62]. This translates into two situations that deviate from what is normally expected in a child’s environment: (1) neglect/deprivation and (2) abuse/trauma [63]. On the other hand, Benzi et al. [64] suggested that abuse and neglect, both physical and emotional, can have a significant impact on a person’s ET, affecting how individuals relate to social information. This can lead to increased doubt (EM) or uncritical acceptance (EC). Emphasising the results obtained considering neglect, Kim and Cicchetti [65] argue that children who experience deviations from the expected environment in the form of neglect or abuse are at risk of a range of maladaptive outcomes due to ineffective emotional regulation, which may explain why trust declines. Adversity can generate long-term disruptions in adaptive capacity, compromising social learning as a result of suspicion and the inability to accurately identify trustworthy sources [2]. Regarding the correlations mentioned in our second hypothesis, only the negative correlation between types of neglect and trust is confirmed, this not being the case with types of abuse. Therefore, this second hypothesis is partially accepted. However, we found that EM and EC are positively associated (with a moderate to strong effect in most correlations) with a higher rate of adverse childhood experiences (abuse, maltreatment, and/or neglect), higher levels of hypomentalisation, symptoms of anxiety and depression, and anxious and fearful avoidant attachment styles. Therefore, our third hypothesis is confirmed.

Finally, with respect to anxious-depressive symptomatology, moderate (EM) and moderate-strong (EC) correlations were found. This is in line with previous studies exploring syntomatology [66,67]. Parolin et al. [66] explored mentalisation and epistemic confidence as protective mechanisms against emotional dysregulation in adolescents with internalising symptoms, also using the RFQ to assess hypomentalisation. Likewise, Riedl et al. [67] found in psychosomatic inpatients receiving rehabilitation treatment that lower mentalisation and higher EM and EC, were associated with higher levels of depression, anxiety and somatization, as well as with lower health-related quality of life (HRQoL). For EM, the strongest positive correlations were found with anxiety, whereas for EC, they were found with depression. In turn, higher ET was associated with lower levels of depression, anxiety, somatization, and most HRQoL subscales. Here, the highest negative correlations were found with impaired social functioning. These authors found, as we did, that the EM and EC factors of the epistemic scale were significantly associated with internalising symptomatology. Our data adds to these findings information about the general population, suggesting that anxious-depressive (internalising) symptoms are directly related to the EM factor and especially to EC. Therefore, taking into account mistrustful stances and especially overconfident ones could be relevant for therapeutic interventions considering that they might show evident internalising symptoms (anxious-depressive) and tend to adopt such epistemic stances. Likewise, it is essential to consider these factors for psychological interventions, since they could have an impact on adherence to treatment and the therapeutic bond.

Our findings support the invariance of the instrument (configural, metric, scalar and residual) between genders, indicating that the structure, the relationship of the items and the measurement scale is equivalent between the groups. Having verified the invariance we compared the mean scores of the two groups revealing that women obtain higher scores in ET, these same results have been obtained in other studies [64,67]; partially similar to [17] and Campbell et al. [2], which found higher ET and EC scores in women in the first study and in the second study only in EM.

Taken together, results of both studies validate the reliability and applicability of the ETMCQ in the Argentine population, which may be useful for clinical and therapeutic purposes. In addition, it suggests the validity of exploring in future research the various roles that ET may have with other constructs, helping to understand more precisely the relationships of this concept in mental health pathology.

There are limitations to the study, including the fact that the variable age has not been controlled for, as it has an extremely wide range (18–89 years). This wide age distribution may affect the representativeness of the instrument, as it is possible that the results are influenced by differences in the age of the participants. Another limitation of this research is that it was conducted in a non-clinical population, which could be highly heterogeneous and introduce a large variability in the data. Likewise, it would have been valuable to include information about mental health diagnoses in the samples of both studies to deepen the explanations of some of the findings. Future studies should consider controlling for or adjusting the mentioned sociodemographic variables to obtain more accurate results. For this reason, the validation of this version of the ETMCQ in a clinical sample is pending for future research to confirm that it is a reliable and valid instrument for this population and, at the same time, to expand and learn about its applicability and usefulness in clinical practice.

In addition, we did not collect exactly the same sociodemographic data between the two studies; the second study had more complete sociodemographic data which prevented a comparison of this information. This differential data collection limits the analyses of our study, as we cannot create a model that includes both samples and review the data jointly.

Another limitation of our study is that it was based exclusively on self-report measures, without incorporating other types of data from different methodologies. The exclusive use of self-reports may introduce biases, such as social desirability, which could affect the accuracy and validity of the results. The inclusion of data from direct observations as clinical assessments, or physiological measures, among others, could provide a more complete and accurate understanding of the reported results.

A final limitation related to the EM factor reminds us that the obtained reliability may be questionable. Although the results in this study have been significant for addressing criterion validity, other studies [17,44,21] have identified the low reliability of items evaluating this factor as a potential major issue. Additionally, Campbell et al. [2] in their Study 2, Liotti et al. [20], and Greiner et al. [21] also reported inadequate or barely acceptable reliability results for this factor. Therefore, it is crucial that future research focuses on ensuring the reliability of this factor and on determining how the ETMCQ performs in other Spanish-speaking countries. Specifically, future studies should assess whether the instrument demonstrates invariance across different cultural contexts to ensure that measurements are consistent and comparable across diverse Spanish-speaking populations.

## Supporting information

S1 Appendix Items from the Argentinian version of the epistemic trust, mistrust, and credulity questionnaire (ETMCQ). (PDF)

S1 Table Dataset 1 from Study 1. (CSV)

S2 Table Dataset 2 from Study 2. (CSV)

S3 Table Dataset 3 from Study 2. (CSV)
