# Supplementary material for: Validation of the Argentine version of the epistemic trust, mistrust, and credulity questionnaire
Source: PLoS One. 2024 Oct 3;19(10):e0311352. doi: 10.1371/journal.pone.0311352 (PMC11449354; doi:10.1371/journal.pone.0311352)
Supplement: S1 Appendix — (PDF) [file pone.0311352.s001.pdf]

Appendix A

Argentinian Version of the Epistemic Trust, Mistrust, and Credulity Questionnaire (ETMCQ)							
Lea las siguientes afirmaciones e indique en qué medida está de acuerdo o en desacuerdo marcando el número que más se corresponde con su opinión y experiencia. Utilice la siguiente escala de valoración, seleccionando 7 si está muy de acuerdo, y 1 si está muy en desacuerdo. El punto medio, si es neutral o indeciso, es 4.							
Ítems	1. Muy en desacuerdo	2. En desacuerdo	3. Un poco en desacuerdo	4. Ni de acuerdo ni en desacuerdo	5. Un poco de acuerdo	6. De acuerdo	7. Muy de acuerdo
1. Usualmente pido consejo a la gente cuando tengo un problema personal.	1	2	3	4	5	6	7
2. Se me hace más fácil confiar y asimilar la información cuando proviene de alguien que me conoce bien.	1	2	3	4	5	6	7
3. Prefiero averiguar por mi cuenta las cosas en internet a tener que pedirle información a otras personas.	1	2	3	4	5	6	7

4. A menudo siento que las personas no entienden lo que quiero y necesito.	1	2	3	4	5	6	7
5. Con frecuencia me consideran ingenuo/a porque creo casi todo lo que me dicen.	1	2	3	4	5	6	7
6. Cuando hablo con otras personas, noto que lo que me dicen me convence fácilmente, incluso si antes creía algo distinto.	1	2	3	4	5	6	7
7. A veces, conversar con personas que me conocen desde hace mucho tiempo me ayuda a desarrollar nuevas perspectivas sobre mí mismo/a.	1	2	3	4	5	6	7
8. Considero muy útil aprender de lo que la gente me cuenta acerca de sus experiencias.	1	2	3	4	5	6	7
9. Si confías demasiado en lo que la gente te dice, es probable que salgas herido/a.	1	2	3	4	5	6	7

10. Cuando alguien me cuenta algo, mi reacción inmediata es preguntarme porqué me están diciendo esto.	1	2	3	4	5	6	7
11. Muchas veces he seguido el consejo de las personas equivocadas.	1	2	3	4	5	6	7
12. La gente me ha dicho que soy muy fácilmente influenciable por otros.	1	2	3	4	5	6	7
13. Si no sé qué hacer, mi primer instinto es consultarle a alguien cuya opinión valoro.	1	2	3	4	5	6	7
14. Usualmente no sigo los consejos de los demás, incluso cuando creo que probablemente son correctos.	1	2	3	4	5	6	7
15. En el pasado he juzgado mal a quien creer, y se han aprovechado de mí.	1	2	3	4	5	6	7
